# Monolithic multiple colour emission from InGaN grown on patterned non-polar GaN

**DOI:** 10.1038/s41598-018-37575-7

**Published:** 2019-01-30

**Authors:** Y. Gong, L. Jiu, J. Bruckbauer, J. Bai, R. W. Martin, T. Wang

**Affiliations:** 1 Department of Electronic and Electrical Engineering, University of Sheffield, Mappin Street, Sheffield, S1 3JD United Kingdom; 2 Department of Physics, SUPA, University of Strathclyde, Glasgow, G4 0NG United Kingdom

## Abstract

A novel overgrowth approach has been developed in order to create a multiple-facet structure consisting of *only non-polar and semi-polar GaN facets without involving any c-plane facets*, *allowing the major drawbacks of utilising c-plane GaN for the growth of III-nitride optoelectronics to be eliminated*. Such a multiple-facet structure can be achieved by means of overgrowth on non-polar GaN micro-rod arrays on *r*-plane sapphire. InGaN multiple quantum wells (MQWs) are then grown on the multiple-facet templates. Due to the different efficiencies of indium incorporation on non-polar and semi-polar GaN facets, multiple-colour InGaN/GaN MQWs have been obtained. Photoluminescence (PL) measurements have demonstrated that the multiple-colour emissions with a tunable intensity ratio of different wavelength emissions can be achieved simply through controlling the overgrowth conditions. Detailed cathodoluminescence measurements and excitation-power dependent PL measurements have been performed, further validating the approach of employing the multiple facet templates for the growth of multiple colour InGaN/GaN MQWs. It is worth highlighting that the approach potentially paves the way for the growth of monolithic phosphor-free white emitters in the future.

## Introduction

The last two decades have seen tremendous progress in developing III-nitride semiconductor visible light emitters for solid-state lighting. III-nitrides all exhibit direct bandgaps, covering the whole visible spectral region from deep ultraviolet to infrared (from 6.2 eV for AlN through 3.5 eV for GaN to 0.7 eV for InN). Therefore, in principle, III-nitrides can be fabricated into white light emitters without involving any other material systems. However, the state-of-the-art achieved so far is still based on the well-known “blue light emitting diodes (LEDs) + yellow phosphor” approach, where the blue emission from InGaN/GaN-based LEDs grown on *c-plane* substrates radiatively pumps yttrium aluminium garnet (YAG) phosphors to provide yellow emission leading to the generation of white light^1–4^. It is worth highlighting that this approach suffers a number of drawbacks, such as the self-absorption of phosphors leading to additional limits on the overall efficiency, the issue on quenching and stability of phosphors as a result of heat generated, etc^5–7^. Furthermore, another fundamental issue of this approach is due to the very slow response time of phosphors, typically on the order of microseconds, significantly limiting a modulation bandwidth down to the 1 MHz level for visible light communication (Li-Fi) applications^8^.

It is expected that an ultimate lighting source for general illumination would be a phosphor-free white LED, which requires both the highest efficiency and the best colour rendering index. It is also required that both colour quality and optical efficiency remain unchanged with increasing injection current. Phosphor-free white LEDs developed so far are primarily grown on *c-plane* substrates, i.e., by introducing InGaN multiple quantum wells (MQWs) with different indium composition as an active region^9–12^. However, due to the well-known polarisation induced quantum confined Stark effect (QCSE), it is difficult to achieve a phosphor-free white LED with high optical efficiency on a *c-plane* substrate^13^. Another great challenge in developing a monolithic white LED is due to the fundamental limitation in incorporating high indium content into GaN on *c-plane* substrates, while InGaN with high indium content is crucial for achieving green or yellow emission, the major components for phosphor-free white LEDs. The QCSE issue also leads to an increase in carrier recombination lifetime, thus significantly reducing the modulation bandwidth if such a white LED is used for Li-Fi. This becomes even worse for longer wavelength such as green or yellow emission.

In summary, it is extremely difficult to achieve phosphor-free white LEDs with desired performance on *c-plane* substrates. Monolithic multi-colour emissions have been reported by means of employing three dimensional (3D) GaN structures including GaN hexagonal annular structures^14–16^. However, these structures still involve the utilization of *c-plane* GaN^9,10,14–18^. Therefore, the fundamental issues still exist.

In order to resolve these challenges, we need to design a new structure with multiple facets consisting of *non-polar and semi-polar facets only*, *but without involving any c-plane facets*. Such requirements cannot be met by using current III-nitride emitters.

It is well-known that a significant reduction in QCSE can be achieved by means of the growth of InGaN LEDs on non- or semi- polar GaN, leading to significantly enhanced internal quantum efficiency^19,20^. As a consequence, this also greatly reduces a carrier recombination lifetime, thus effectively increasing a modulation bandwidth for Li-Fi. Second, indium incorporation efficiency can be significantly enhanced if InGaN is grown on semi-polar GaN such as (11–22) GaN in comparison with its *c-plane* counterparts, which is crucial for the growth of longer wavelength such as yellow/amber/red LEDs^20–22^. Thirdly, non-polar GaN generally leads to reduced indium incorporation efficiency (if InGaN is grown on its top) in comparison with its *c-plane* counterpart. Therefore, under identical growth conditions, InGaN MQWs grown on semi-polar and non-polar facets will exhibit different wavelength emissions in a single chip if the wafer consists of semi-polar and non-polar facets, potentially obtaining white lighting without any concerns about the drawbacks resulting from the growth on *c-plane* facets. In order to address this issue, we are proposing a multiple-facet structure which consists of only non-polar GaN facet and semi-polar GaN facets in a single chip, where the InGaN MQWs grown on the non-polar GaN surface is used as a short wavelength emitting region and the InGaN MQWs on the semi-polar GaN surface as a long wavelength emitting region. This potentially leads to monolithic multiple-colour lighting but without involving any growth on a *c-plane* surface.

Two major challenges need to be overcome in order to achieve the above idea. One is due to the crystalline quality of current non- or semi-polar GaN on sapphire or silicon which is far from satisfactory. Another is how to achieve a multiple facet structure without forming *c-plane* GaN facets.

In this work, we present a novel overgrowth approach to create a multiple-facet structure consisting of *only non-polar and semi-polar GaN facets without involving any c-plane facets* by means of overgrowth on non-polar GaN micro-rod arrays on *r-plane* sapphire. Such overgrowth on the micro-rod arrayed templates on sapphire substrates provides an effective approach to achieving significantly improved crystal quality. For details, please refer to ref.^23^. The dislocation density of our non-polar GaN overgrown on micro-rod arrayed templates has been substantially reduced down to 6 × 10^8^/cm^2^ from a typical dislocation density of 10^11^/cm^2^ for non-polar GaN directly grown on sapphire without involving overgrowth. As a validation purpose, InGaN MQWs are then grown on the multiple-facet structure. Both photoluminescence (PL) and cathodoluminescence (CL) measurements have demonstrated multiple-colour emissions with a tuneable intensity ratio of different wavelength emissions which can be achieved simply through controlling overgrowth time.

Standard non-polar (11–20) GaN is initially grown on *r-plane* sapphire by means of a standard metalorganic vapour phase epitaxial (MOVPE) technique using our high temperature AlN buffer approach^24^. The non-polar GaN is then fabricated into regular micro-rod arrays with a SiO_2_ mask on top of each micro-rod. The details of the fabrication procedure can be found elsewhere^23^.

**Figure 1:**
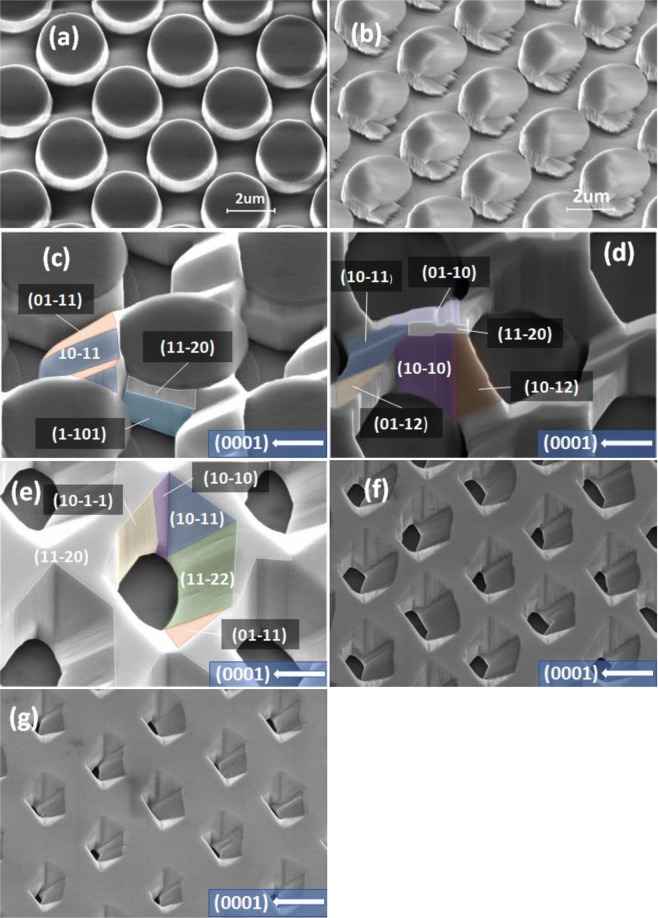
(**a**) Plan-view SEM image of our non-polar GaN micro-rod arrays; (**b**) Plan-view SEM image of our non-polar GaN micro-rod arrays with a “mushroom” configuration; (**c**–**g**) Plan-view SEM images of overgrown GaN on the non-polar GaN micro-rod arrays with a “mushroom” configuration as a function of overgrowth time of 1000, 2000, 3000, 3500 and 4000 seconds, respectively, where multiple semi-polar and non-polar facets have been marked.

Figure 1a shows a plan-view scanning electron microscopy (SEM) image of our regularly arrayed micro-rod template in a chessboard configuration, demonstrating that the diameter of micro-rods is 2.5 μm. Furthermore, dry etching is performed down to the sapphire substrate.

The micro-rod template further undergoes an ultraviolet light assisted photochemical etching process in a 10% KOH solution under an illumination of a Xenon lamp with a power density of 1.5 W/cm^2^, forming a “mushroom” configuration as shown in Fig. 1b. Specifically, the side of each micro-rod that faces in the <000–1> direction is etched, while this process does not etch the SiO_2_ masks at all. This process does not etch GaN surfaces facing in the <0001> direction, either, but does effectively etch GaN facing in the <000–1> direction. With such a “mushroom” configuration, lateral growth along the <000–1> direction (i.e., -*c* orientation) can be effectively suppressed. This not only effectively reduces defects but also helps to form semi-polar facets. Such a regularly arrayed micro-rod template was originally designed for overgrowth of (11–20) non-polar GaN with a step-change in crystal quality^23^.

Finally, the non-polar GaN micro-rod array template is reloaded into the MOVPE chamber for further overgrowth. A schematic illustration is provided in Supplemental Information in order to demonstrate the formation processes of a multiple-facet structure by means of our overgrowth on the regularly arrayed non-polar GaN micro-rod templates discussed above.

Figure 1c–g show the SEM images of an evolution of overgrown non-polar (11–20) GaN on the above micro-rod arrays as a function of overgrowth time of 1000, 2000, 3000, 3500 and 4000 sec, respectively, demonstrating the formation of a multiple facet structure consisting of non-polar and semi-polar GaN facets. It can be observed that lateral growth from the <0001> direction can be observed during the first coalescence process (0–2000 seconds), while lateral growth along the <000–1> orientation is suppressed as a result of the “mushroom” configuration. In the meantime, multiple semi-polar facets naturally form during the first coalescence process. During the second coalescence process (2000–4000 seconds), where the lateral growth of GaN extends above the SiO_2_ masks, multiple GaN facets including non-polar (11–20), semi-polar (11–22), (01–11), (10–11) and (1–101) facets labelled in Fig. 1e have been formed. Please note that there are no *c-plane* facets formed on such a micro-rod arrayed non-polar GaN template.

With increasing overgrowth time, the area of non-polar (11–20) facet which is the top surface starts to increase. When the overgrowth time reaches 4000 sec, the template is still un-coalesced but the non-polar (11–20) facet dominates the structure. The formed arrayed micro holes as a result of the un-coalescence lead to the formation of semi-polar facets, among which the (11–22) facet exhibits the largest area. Clearly, by controlling overgrowth time, the facet area ratio of semi-polar to non-polar GaN can be tuned, which in return determines the ratio of emission intensity of long wavelength emission to short wavelength emission once InGaN MQWs are grown on top.

In order to confirm the above idea, an InGaN/GaN MQW structure with 3 periods has been grown under identical conditions on the three templates obtained using different overgrowth time, namely 3000, 3500 and 4000 sec, which are labelled as sample A, sample B and sample C, respectively. All the samples end with a 100 nm GaN cap layer. Figure 1c–g clearly show that the facet area ratio of semi-polar to non-polar GaN is determined by controlling overgrowth time. The facet area ratio is reduced by increasing overgrowth time. PL measurements have been performed on the three samples.

**Figure 2:**
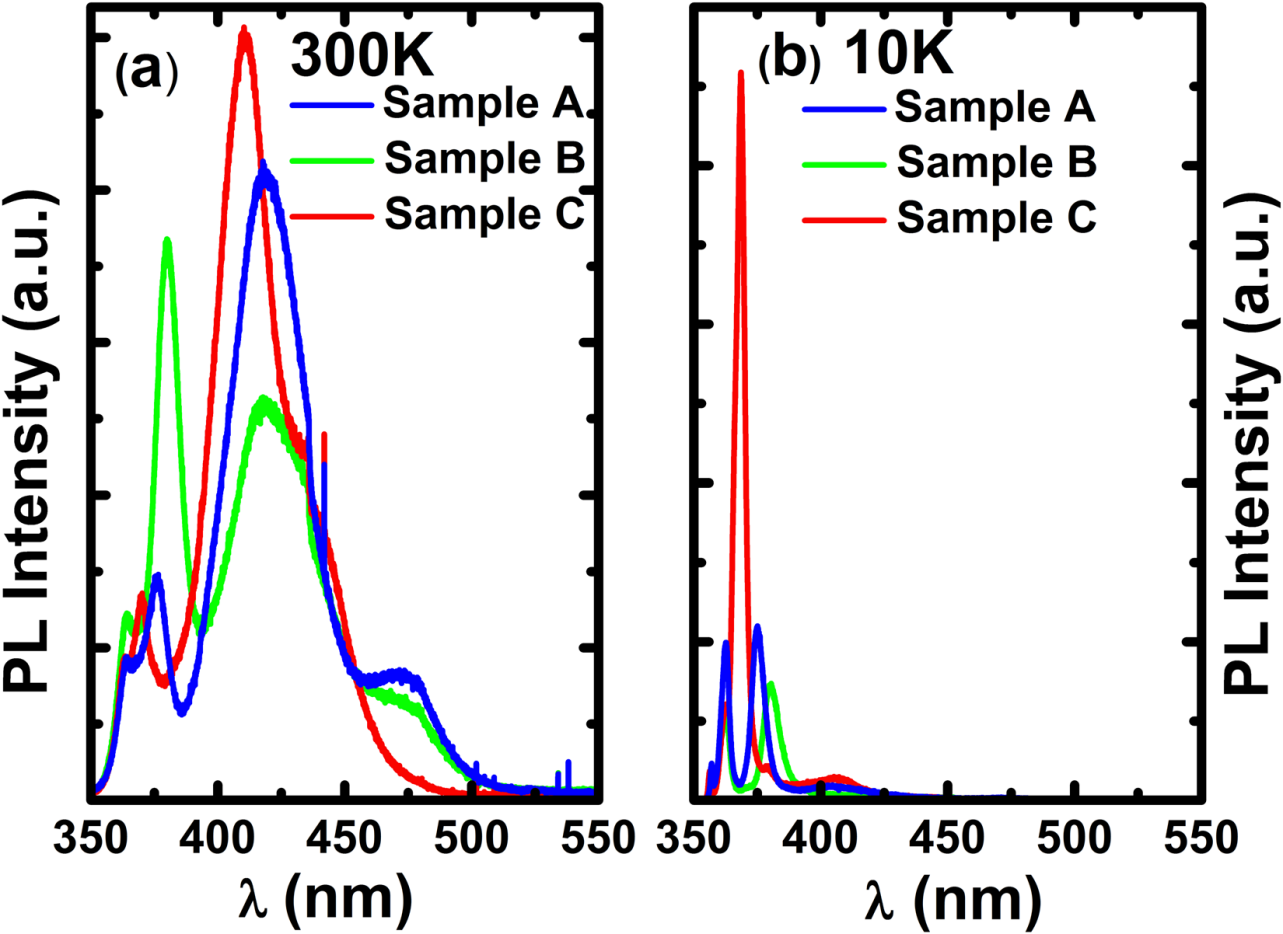
PL spectra of sample A, B and C, measured at room temperature (**a**,**b**) 10 K using a 325 nm He-Cd laser.

Figure 2a show the PL spectra of the three samples, measured by using a 325 nm He-Cd laser under an excitation power density of 100 W/cm^2^ at room temperature (RT). In each case, there are three peaks present, one at 362 nm which is from the GaN layer, one around 380 nm, and another one around 410–420 nm. Figure 2b displays the PL spectra of these samples measured at 10 K also using the 325 nm He-Cd laser.

By examining the RT PL spectra as shown in Fig. 2a very carefully, for sample A and sample B, there exists an additional peak at ~ 475 nm. By detailed comparison, the peak at 475 nm in sample A shows higher intensity than that in sample B. These emissions below the GaN bandgap are from the InGaN MQWs grown on either the (11–20) non-polar facet or the semi-polar facets within the micro-holes formed as a result of overgrowth as shown in Fig. 1.

In order to identify the correspondence between these emission peaks and the multiple-facets, detailed CL measurements have been performed on the three samples using CL hyperspectral imaging^25^. The sample is placed at an angle of 45° with respect to the incident electron beam. The projection of the <0001> direction, i.e., +c direction, is pointing downward in the CL images.

**Figure 3:**
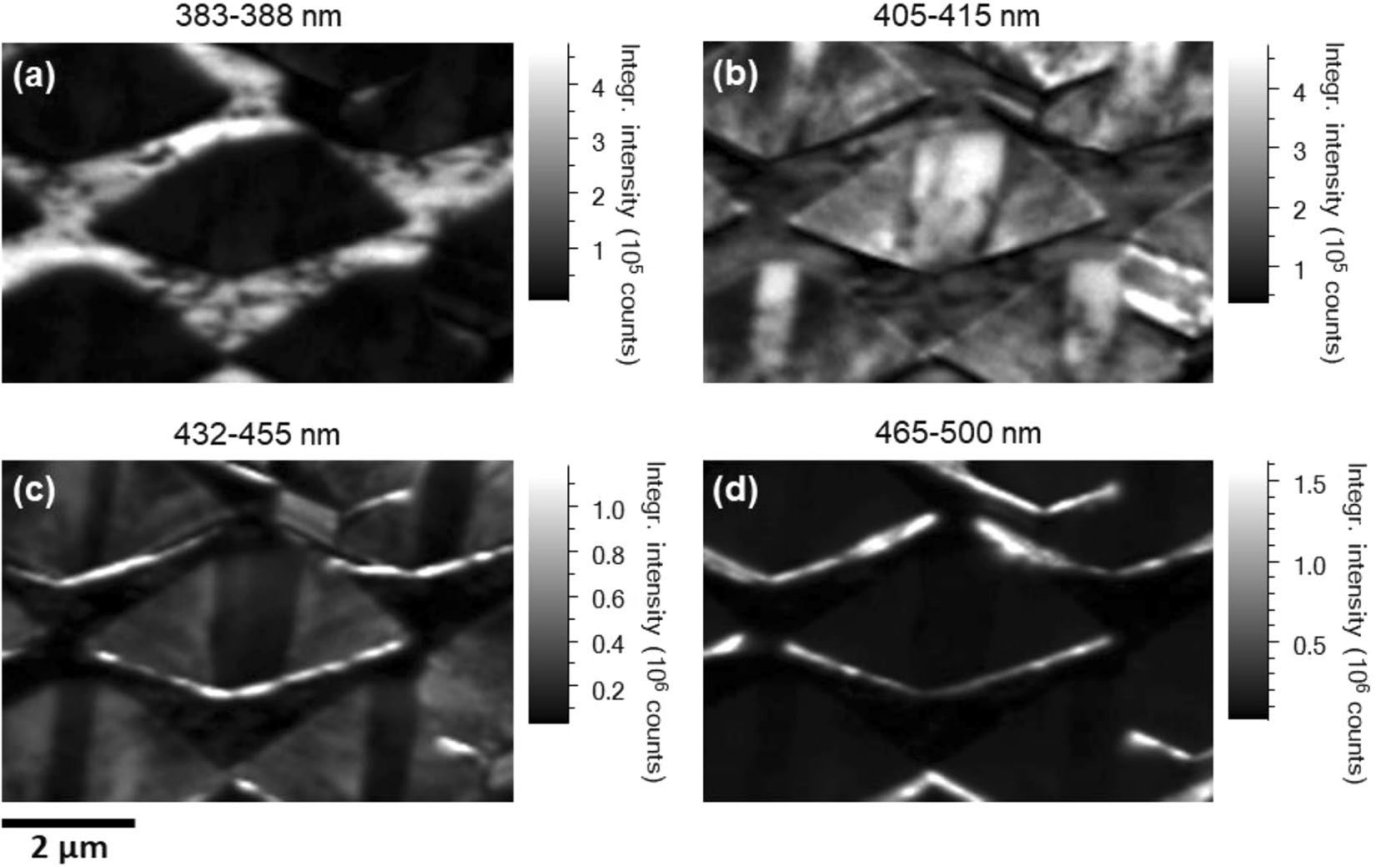
Room temperature integrated CL intensity images obtained at 5 kV of sample B calculated for the following spectral ranges: (**a**) 383–388 nm, (**b**) 405–415 nm, (**c**) 432–455 nm and (**d**) 465–500 nm. The images do not account for the 45° tilt of the sample, and thus the y-axis in each case will need to be corrected by a factor of 1.41.

As a typical example, Fig. 3a–d show the integrated CL intensity images of sample B calculated from the hyperspectral data set obtained at 5 kV for four spectral ranges of 383–388 nm, 405–415 nm, 432–455 nm and 465–500 nm, respectively, focusing on the InGaN MQWs grown on the different facets. Owing to the higher spatial resolution compared with the area averaged PL measurements in Fig. 2, the CL measurement allows us to identify four different emission peaks associated with the MQWs grown on the four different facets.

The 383–388 nm emission is from the top flat surface, which is from the InGaN MQWs grown on the (11–20) non-polar facet as expected. The 405–415 nm emission is due to the InGaN MQWs grown on the semi-polar (11–22) facet (i.e., the semi-polar facet with the largest area within the micro-hole formed as shown in Fig. 1e**)**. The emission at 432–455 nm is primarily from the (10–11) and (01–11) semi-polar facets, while the 465–500 nm emission peak is from the (10–1–1) and (01-1-1) semi-polar facets. It is well-known that InGaN grown on different semi-polar facets exhibit different indium incorporation efficiency, leading to emission with different wavelengths. The results further confirm that indium incorporation efficiency of InGaN on semi-polar GaN facets exhibits: (10-1-1) > (10–11) > (11–22), which is in good agreement with the previous studies^20^. All the semi-polar facets exhibit higher indium incorporation efficiency than the non-polar (11–20) facet, also matching the previous studies very well^17,18,26^.

Comparing the RT PL of the three samples, the emission intensity ratio of short wavelength emission (non-polar) to long wavelength emissions (semi-polar) is different, as the non-polar facet area increases and the multiple semi-polar facet area decreases with increasing overgrowth time. It is worth highlighting that both the (11–20) nonpolar facet and the semi-polar facets with well-controlled areas can be achieved, potentially allowing us to tune the ratio of the emission intensities of InGaN MQWs grown on these facets.

It is well-known that the radiative recombination lifetime of InGaN MQWs is very short at a low temperature, typically hundreds of picoseconds, where the radiative recombination processes at a low temperature dominate the emission. Therefore, if a 325 nm He-Cd laser is used as an excitation source, the emission from GaN and the emission from non-polar InGaN MQWs at 380 nm can be observed, while it would be difficult to observe the emission from any semi-polar GaN MQWs (where the InGaN content is higher than that in the non-polar InGaN MQWs). The physics behind is due to the fact that the radiative recombination lifetime of InGaN MQWs is so short that there is not enough time for excitons to diffuse to the semi-polar InGaN MQWs. This is what has been observed in Fig. 2b.

However, if a 375 nm laser is used as an excitation source, the emissions from the semi-polar InGaN MQWs can be observed as the non-polar InGaN MQWs at 380 nm are not optically excited considering that there may be a Stokes-shift^20^. Of course, at room temperature, it is well-known that non-radiative recombination processes dominate the emission of InGaN/GaN MQWs. As a result, the emission from semi-polar InGaN/GaN MQWs can be observed regardless of whether a 325 nm laser or 375 nm laser is used as an excitation source.

**Figure 4:**
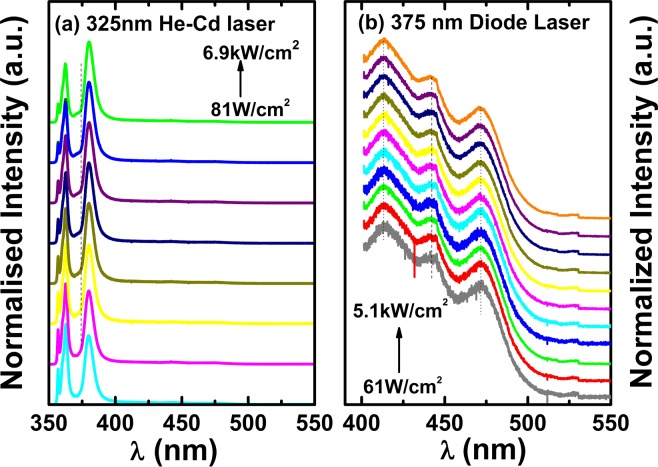
Excitation power density dependent PL spectra of sample B measured at 10 K using a 325 nm He-Cd laser (**a**); and a 375 nm diode laser (**b**).

Figure 4a shows the excitation power dependent PL spectra of sample B measured at 10 K using a 325 nm He-Cd laser, indicating that there are only three peaks present with increasing excitation power density from 81 W/cm^2^ to 69 kW/cm^2^. The peaks at 357 nm and 362 nm are due to the near-band edge emission (NBE) of GaN and the GaN basal-plane stacking fault related emission, respectively, which have been well-studied^27^. The peak at 380 nm is due to the non-polar InGaN MQWs, and there is no blue-shift observed with increasing excitation power density, a typical finger print for non-polar InGaN MQWs, which do not exhibit the QCSE. These results agree well with our expectation stated above.

In order to investigate the optical properties of the InGaN/GaN MQWs from semi-polar facets, a 375 nm laser diode is used as an excitation source, where the non-polar MQWs cannot be excited. Figure 4b shows the PL spectra as a function of excitation power density from 61 W/cm^2^ to 5.1 kW/cm^2^ measured at 10 K. The emission peaks around 413 nm, 442 nm, and 472 nm are from the MQWs on the different semi-polar facets as discussed above. In contrast to the non-polar (11–20) emission peak, these emission peaks at 413 nm, 442 nm and 472 nm exhibit a weak blueshift of up to 1 nm in emission wavelength with increasing excitation power density, a typical behaviour for semi-polar InGaN MQWs. Of course, the blueshift is much weaker than that for their *c*-plane counterparts, agreeing with significantly reduced QCSE in comparison with their *c*-plane counterparts.

In conclusion, we have reported a novel approach to grow a multiple-facet structure consisting of *only non-polar and semi-polar GaN facets without involving any c-plane facets* by means of overgrowth on a non-polar GaN micro-rod array template on *r*-plane sapphire. Multiple-colour InGaN/GaN MQWs grown on such a multiple facet template have been achieved. Both PL and CL measurements have confirmed that the longer wavelength emission is from the InGaN/GaN MQWs grown on semi-polar facets, while the shorter wavelength emission is from the MQWs grown on the (11–20) non-polar facet. This is due to higher indium incorporation efficiency of InGaN grown on a semi-polar facet in comparison with that grown on a non-polar facet. The emission intensity ratio of the longer wavelength emission to the shorter wavelength emission can be tuned by simply controlling the thickness of the multiple-facet templates. The presented approach has demonstrated a great potential to achieve a white phosphor-free LED with high performance.

## Methods

### Fabrication of regular microrod arrays

A SiO_2_ film with a thickness of 150 nm is first deposited on the as-grown non-polar GaN using a standard plasma enhanced chemical vapour deposition (PECVD) technique. Subsequently, by means of a standard photolithography approach and then a reactive ion etching (RIE) technique, the SiO_2_ film is fabricated into regularly arrayed micro-rods with a micro-rod diameter of 2.5 μm. The regularly arrayed SiO_2_ micro-rod arrays are then used as a second mask to finally etch the GaN layer underneath into regular GaN micro-rod arrays by using a standard inductively coupled etching (ICP) system. The GaN etching is performed down to the sapphire substrate. Each SiO_2_ micro mask formed remains on top of each GaN micro-rod. Subsequently, an ultraviolet light assisted photochemical etching process in a KOH solution have been performed, finally forming a “mushroom” configuration as a result of selective etching between N-face GaN and Ga-face GaN.

Photoluminescence measurements have been carried out by using either a commercial 325 nm He-Cd laser or a 375 nm diode laser as the excitation source. The emission is dispersed by a monochromator (Horiba SPEX 500 M) and then detected by an air-cooled charge-coupled device (CCD). The samples are placed in a closed*-*cycle helium cryostat, where the temperature range can be controlled from 10 to 300 K.

Cathodoluminescence hyperspectral imaging was carried out at room temperature using a variable pressure field emission scanning electron microscope (FEI Quanta 250). The emitted light was collected by a Cassegrain reflecting objective, focussed on the entrance slit of a 1/8 m focal length spectrometer (Oriel MS125) and detected using an electron multiplying charge-coupled device (Andor Newton). The CL was collected in hyperspectral imaging mode, meaning a full CL spectrum was collected per pixel in an image^28^.

## Supplementary information

Supplementary info
