# Supplementary material for: Monolithic multiple colour emission from InGaN grown on patterned non-polar GaN
Source: Sci Rep. 2019 Jan 30;9:986. doi: 10.1038/s41598-018-37575-7 (PMC6353934; doi:10.1038/s41598-018-37575-7)

Monolithic multiple colour emission from InGaN grown on patterned non-polar GaN

Y. Gong[1], L. Jiu[1], J. Bruckbauer[2], J. Bai[1], R. W. Martin[2] and T. Wang[1,*]

[1]Department of Electronic and Electrical Engineering, University of Sheffield, Mappin Street, Sheffield S1 3JD, United Kingdom

[2]Department of Physics, SUPA, University of Strathclyde, Glasgow G4 0NG, United Kingdom

*E-mail: t.wang@sheffield.ac.uk

Supplementary Information

Our multiple-facet structures are formed by means of overgrowth on regularly arrayed non-polar GaN micro-rod templates which undergo selective photo-chemical etching processes in a 10% KOH solution under an illumination of a Xeon lamp with a power density of 1.5 W/cm^2. Afterwards, such a non-polar GaN micro-rod array template is reloaded into the MOVPE chamber for further overgrowth. **Figure S1** provided below schematically illustrates the whole process, corresponding to Figure 1 shown in our manuscript: (a) It starts with non-polar GaN micro-rod arrays as described in the manuscript; and (b) forms a “mushroom” configuration by selective etching non-polar GaN micro-rods along the <000-1> direction; and then (c) - (f) laterally grows from the <0001> direction during the first coalescence process (0-2000 seconds), while lateral growth along the <000-1> orientation is suppressed as a result of the “mushroom” configuration. During the 2nd coalescence process (2000-4000 seconds), where the lateral growth of GaN extends above the SiO_2 masks, multiple GaN facets including non-polar (11-20), semi-polar (11-22), (01-11), (10-11) and (1-101) facets labelled in Figure 1e in the manuscript have been formed. The facet area ratio of semi-polar to non-polar GaN is determined by controlling overgrowth time.

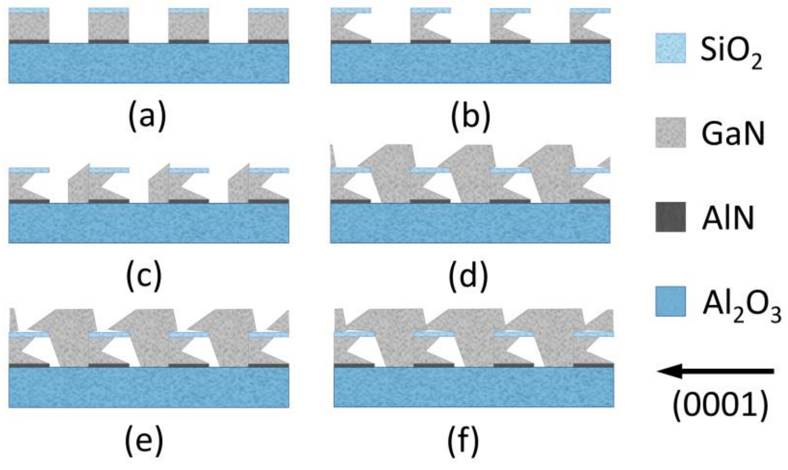

Supplement: Supplementary file 1 — Supplementary info [file 41598_2018_37575_MOESM1_ESM.pdf]
